# Establishment of a translational endothelial cell model using directed differentiation of induced pluripotent stem cells from Cynomolgus monkey

**DOI:** 10.1038/srep35830

**Published:** 2016-10-25

**Authors:** Eva C. Thoma, Tobias Heckel, David Keller, Nicolas Giroud, Brian Leonard, Klaus Christensen, Adrian Roth, Cristina Bertinetti-Lapatki, Martin Graf, Christoph Patsch

**Affiliations:** 1 Roche pRED (Pharmaceutical Research and Early Development), Roche Innovation Center Basel, F. Hoffmann-La Roche Ltd., Basel, Switzerland

## Abstract

Due to their broad differentiation potential, pluripotent stem cells (PSCs) offer a promising approach for generating relevant cellular models for various applications. While human PSC-based cellular models are already advanced, similar systems for non-human primates (NHPs) are still lacking. However, as NHPs are the most appropriate animals for evaluating the safety of many novel pharmaceuticals, the availability of *in vitro* systems would be extremely useful to bridge the gap between cellular and animal models. Here, we present a NHP *in vitro* endothelial cell system using induced pluripotent stem cells (IPSCs) from Cynomolgus monkey (Macaca fascicularis). Based on an adapted protocol for human IPSCs, we directly differentiated macaque IPSCs into endothelial cells under chemically defined conditions. The resulting endothelial cells can be enriched using immuno-magnetic cell sorting and display endothelial marker expression and function. RNA sequencing revealed that the differentiation process closely resembled vasculogenesis. Moreover, we showed that endothelial cells derived from macaque and human IPSCs are highly similar with respect to gene expression patterns and key endothelial functions, such as inflammatory responses. These data demonstrate the power of IPSC differentiation technology to generate defined cell types for use as translational *in vitro* models to compare cell type-specific responses across species.

In biomedical research, non-human primates (NHPs) offer great promise as models for many aspects of human health and disease. They play a unique role in translational science by bridging the gap between basic and clinical investigations due to their high genetic similarities, similar anatomies, and similar physiologies to humans^1^,^2^,^3^,^4^. Therefore, NHPs are often deemed to be the only relevant species, not only for performing basic research but also for drug development, especially for studying biopharmaceuticals, such as therapeutic antibodies. Thus, the differences in the immune systems between primates and other animals renders NHPs better translational models for studying the mechanism of action, bio-distribution, efficacy and safety of novel biopharmaceuticals^5^. Often, animal studies should be supported by *in vitro* investigations using human and animal cells to determine the relative potency of antibodies in humans and the chosen animal model and to examine specific aspects of antibody safety^6^.

The long term goal of both pharmaceutical and basic research is to reduce animal experimentation to a minimum. Many efforts are dedicated to the development of alternative toxicological tests and models, not only for the increasing ethical and public concerns regarding animal testing^7^ but also to reduce costs, time and logistic constraints that are associated with animal studies in general and, in particular, with NHP assays. Moreover, translatability from NHP studies to humans is not always as accurate as necessary. Although NHPs represent the most suitable species regarding several physiological aspects for predicting human relevant toxicities, as illustrated in the TGN1412 case, there are important inter-species differences that might lead to failures in preclinical safety assessment^8^. For these reasons, the availability of predictive NHP *in vitro* systems would be highly beneficial to fill current gaps in research. Such models would not only allow for a reduction of animal experiments but also provide a platform for the preselection of drug candidates for target engagement and cross-species activity.

Induced pluripotent stem cells (IPSCs) from NHPs^9^,^10^,^11^ offer a promising approach for the establishment of such *in vitro* models because of their broad differentiation potential and their unlimited proliferation capacity. Furthermore, as IPSCs can be derived from any donor, they offer the possibility to generate *in vitro* models from various individuals to represent the genetic variability in a population. The most important advantage of implementing NHP IPSCs as a source for *in vitro* studies may be the fact that corresponding human cells can be derived by similar approaches, thereby allowing for direct inter-species comparison.

Here, we established an endothelial *in vitro* system using IPSCs from Cynomolgus monkey (Macaca fascicularis). Forming the inner layer of blood vessels, endothelial cells are involved in numerous important functions, such as angiogenesis or inflammation and associated disorders, e.g., atherosclerosis. Importantly, they also constitute the barrier between the blood system and other tissue and therefore play a crucial role in drug uptake; they are also often involved in adverse drug reactions, such as drug-induced inflammatory responses^12^.

Endothelial cells arise from the mesoderm, which is specified from the posterior primitive streak during embryogenesis^13^. It has been shown that mimicking of these lineage specification cues allows for the efficient generation of endothelial cells from pluripotent stem cells. While several protocols have been established for human and mouse PSCs^13^,^14^, similar approaches for NHPs are still lacking.

In the current study, we establish an efficient approach to differentiate endothelial cells from monkey IPSCs under chemically defined conditions. The resulting cells show typical endothelial marker expression and functions and, importantly, are highly similar to the corresponding human IPSC-derived endothelial cells. Thus, they represent a valuable system for evaluating endothelial function in NHPs *in vitro* with the option to perform direct comparisons to human equivalent models^14^.

## Results

### Differentiation of Cynomolgus monkey IPSCs into endothelial cells

Induced pluripotent stem cells from Cynomolgus monkey (cIPSCs) were cultured under feeder-free chemically defined conditions^15^. The pluripotent status of the cells was confirmed by immunostaining for OCT4, SOX2, and NANOG and G-banding revealed a normal karyotype (Supplementary Figure S1).

**Figure 1:**
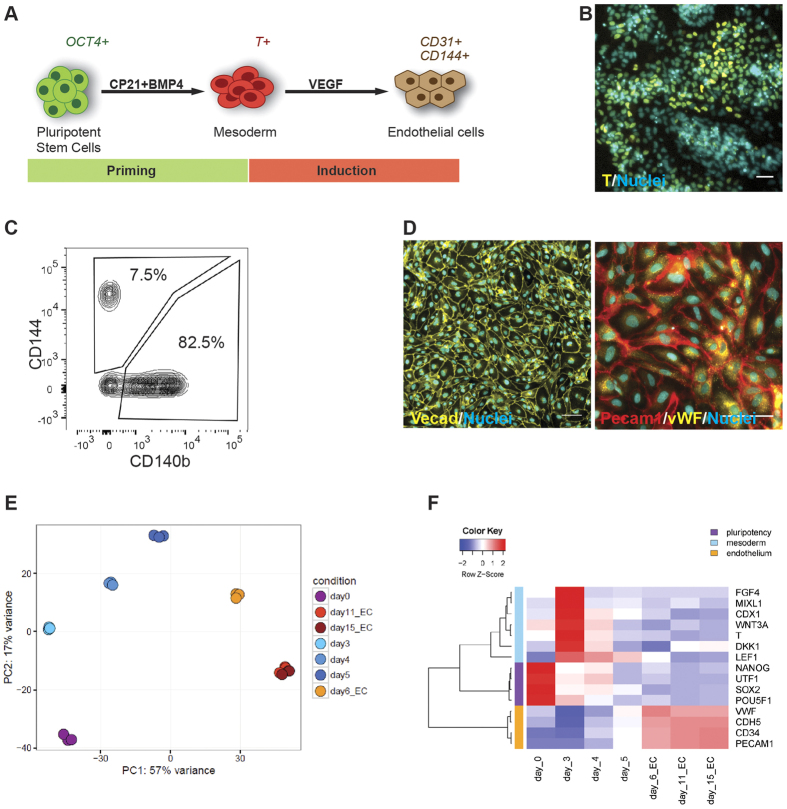
Endothelial differentiation of monkey IPSCs. (**A**) Schematic illustration of the differentiation method. Cynomolgus monkey induced pluripotent stem cells (cIPSCs) were induced to mesoderm commitment (Priming phase) and further differentiated into endothelial cells (Induction phase). (**B**) Immunostaining at day 3 of differentiation indicates the formation of a mesodermal intermediate stage, as shown by the expression of Brachyury/T. Scale bar: 50 μm. (**C**) Flow cytometry analysis at day 6 of differentiation reveals that approximately 7.5% of cells had differentiated into endothelial cells (CD144+), while 82.5% of cells formed vascular smooth muscle cells (CD140b+). (**D**) Immunostaining of cIPSC-ECs at day 13 of differentiation reveals expression of EC specific markers VECAD, PECAM1, and vWF. Scale bars: 100 μm. (**E,F**) Global transcriptome analysis during the differentiation of cIPSCs to endothelial cells. (**E**) Principal component projections of Cynomolgus monkey transcriptomes colored by differentiation time. The variability of the data set along principal component 1 is 57% and along principal component 2 is 17%. (**F**) Heat map of marker gene panels for pluripotency, mesoderm (including Wnt target genes), and endothelial cells. Rows represent genes, and columns represent Cynomolgus monkey cells at different differentiation time points. Row Z-score transformation was performed on mean log_2_ values (n = 3 replicates) for each gene, with blue denoting lower and red denoting higher expression levels compared to the average expression level. Hierarchical clustering of genes and samples is based on complete linkage and Pearson correlation distance.

To differentiate cIPSCs toward the endothelial lineage, we attempted to use a method previously established in our laboratory for human pluripotent stem cells^14^. This approach allows for robust and efficient generation of endothelial cells within six days under chemically defined conditions. Briefly, mesoderm formation is induced during the priming step by activation of BMP and Wnt signaling pathways. In the induction step, the resulting intermediate mesodermal cells are further directed toward the endodermal cell lineage by VEGF and forskolin (Fig. 1A).

We treated cIPSCs with BMP4 and CP21, a novel GSK3β inhibitor, and analyzed mesoderm commitment by immunostaining for the mesodermal marker Brachyury (T). At day 3 of treatment, a majority of the cells stained positive for Brachyury, indicating successful mesoderm commitment (Fig. 1B). The cells were subsequently treated with VEGF and forskolin for two days and analyzed for the expression of the endothelial marker VE-cadherin (CD144, CDH5) and for the vascular smooth muscle cell marker PDGFRβ (CD140b). Flow cytometry analysis showed that approximately 7.5% of cells had formed endothelial cells (CD144+/CD140b−), and 82.5% of cells had formed vascular smooth muscle cells (CD144-/CD140b+) (Fig. 1C). To improve the differentiation efficiency, we tested the effects of cell seeding density, CP21 concentration, and duration of priming step on endothelial differentiation (Supplementary Figure S2A). Quantification of CD144+/CD140b− cells revealed that a CP21 concentration of 1 μM clearly resulted in higher efficiencies than 0.5 μM. Furthermore, a priming period of three days led to higher percentages of endothelial cells, as did initial seeding densities of 45,000 cells/cm^2^. Similar efficiencies were observed when the percentage of endothelial cells was determined using another endothelial marker, PECAM1 (CD31). For this reason, we defined the optimal differentiation conditions as three days of priming, 1 μM CP21, and a seeding density of 45,000 cells/cm^2^, which led to efficiencies of up to 15%. Importantly, this protocol could be reproduced with a different cIPSC lines at comparable efficiencies (Supplementary Figure S2B).

Many applications of *in vitro* models require high purities of one cell type. Therefore, we applied magnetic-activated cell sorting (MACS) to enrich the content of cIPSC-derived endothelial cells, a strategy that has also been successfully applied to human cells^14^. Using this technique, we obtained more than 80% purity of endothelial cells (Supplementary Figure S2C). The purified cells could be expanded, and they maintained the characteristic endothelial morphology and expression of VE-cadherin, PECAM1, and vWF for four passages (Fig. 1D).

To analyze the differentiation process of cIPSCs into endothelial cells in more detail, we performed transcriptome-wide gene expression profiling by RNA sequencing at day 0, day 3, day 4, day 5, day 6, day 11 and day 15 of differentiation using CD144 positive cells from day 6 onwards. For global visualization of the data, we used principal component analysis (PCA). A projection of the complete expression profiles onto the first two principal components revealed that, over the time-course of differentiation, cells became gradually more distinct from IPSCs (Fig. 1E). A time course expression analysis of representative genes for pluripotency, mesoderm, and endothelium showed a rapid downregulation of pluripotency markers followed by a transient upregulation of mesodermal genes, including Wnt target genes, at day 3. Subsequently, endothelial marker genes were upregulated, confirming the commitment toward the endodermal lineage (Fig. 1F). This transition of IPSCs via an embryonic developmental intermediate step toward endothelial cells was further confirmed by unsupervised hierarchical clustering of differentially expressed biological processes obtained from the gene set enrichment analysis (Supplementary Figure S2D). These findings suggest that the *in vitro* differentiation of cIPSCs is highly similar to embryonic vasculogenesis. Strikingly, a similar dynamic signature was observed for human IPSCs (hIPSCs) subjected to this differentiation method^14^, confirming the high similarity of monkey and human IPSCs in their responses to differentiation cues.

### Characterization of cIPSC-derived endothelial cells

**Figure 2:**
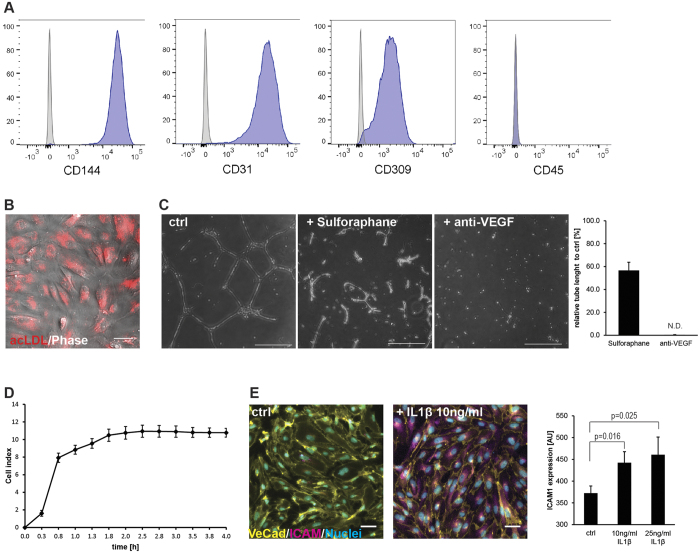
Functional characterization of monkey IPSC-ECs. (**A**) Flow cytometry analysis of cIPSC-ECs at day 13 of differentiation. Cells express endothelial markers CD144, CD31 and CD309 and stain negative for hematopoietic lineage marker CD45. Unstained control samples are shown in grey. (**B**) Uptake of acetylated LDL by cIPSC-ECs. Overlay of phase contrast image and fluorescent channel shows incorporated acLDL (conjugated to Alexa488) in ECs. Scale bar: 50 μm. (**C**) Angiogenic potential of cIPSC-ECs demonstrated by tube formation assay. After 24 hours, cells form a network of tubular structures. Tube formation is inhibited in the presence of 10 μM sulforaphane or 2 μg/ml anti-VEGF antibody. Scale bars: 200 μm. Quantification of the inhibitory effect of anti-angiogenic molecules on the tubulogenesis of cIPSC-ECs (right panel). Columns show total tube length relative to control from three independent experiments. (**D**) Impedance-based monitoring of cIPSC-EC culture demonstrating the formation of a tight monolayer. One of 2 independent experiments performed is depicted (n = 4 technical replicates), error bar STD. (**E**) Response to proinflammatory stimuli. Twenty-four hours after stimulation with proinflammatory cytokines, cIPSC-ECs exhibit upregulated EC. activation marker ICAM1. Scale bars: 50 μm. Quantification of median intensity of ICAM1 staining (right panel). Columns show mean +/− STD of three independent experiments and data were analyzed using Student’s t-test.

To confirm that cIPSC-derived endothelial cells (cIPSC-ECs) display characteristic physiological *in vitro* properties of ECs and are thus applicable as a cellular model, we probed the cells for their marker protein expression, angiogenesis, barrier formation, uptake of lipids, and response to proinflammatory stimuli. Flow cytometry revealed that cIPSC-ECs expressed the typical endothelial surface markers CD144, CD31, and CD309 (KDR) and stained negative for the hematopoietic lineage marker CD45 (Fig. 2A). Furthermore, the cells were able to uptake acetylated LDL, another key function of endothelial cells (Fig. 2B).

Next, the cells were subjected to a tube formation assay to analyze their angiogenic potential. Within 24 hours, cIPSC-ECs formed vascular network-like structures similar to previous studies using endothelial cells from NHP embryonic stem cells^16^,^17^. This process could greatly be perturbed by angiogenesis inhibitors, such as sulforaphane and an anti-VEGF monoclonal antibody^18^ (Fig. 2C).

To determine the tightness of cell-cell contacts of cIPSC-ECs as a parameter for barrier function, we measured impedance using an xCeLLigence RT-CA system^19^,^20^,^21^, which enables real-time monitoring of cell adhesion, viability, and density-dependent proliferation. After the initial attachment, a strong increase in impedance was observed, indicating the formation of a stable continuous endothelium within three hours of plating (Fig. 2D).

Endothelial cells play crucial roles in inflammation processes, as they respond to proinflammatory stimuli with the upregulation of cell adhesion molecules such as ICAM1, enabling the attachment of leukocytes to the endothelium and ultimately their migration to the surrounding tissue. To test if cIPSC-ECs display these functional features, the cells were challenged with the proinflammatory cytokine IL1β. Immunostaining revealed that both treatments induced a strong upregulation of ICAM1, further confirming the endothelial-specific functionality of cIPSC-ECs (Fig. 2E).

### Comparison of monkey IPSC-ECs with human ECs

A major challenge in research using animal models is the question of to what extent findings of animal studies can be translated to humans, e.g., evaluating the clinical relevance of a preclinical toxicity finding in drug development. A promising strategy to address this question is the use of predictive *in vitro* systems from different species. To analyze if monkey IPSC-ECs can be applied to such inter-species comparisons, we sought to determine their similarity to human endothelial cells in terms of gene expression characteristics and key functional features.

**Figure 3:**
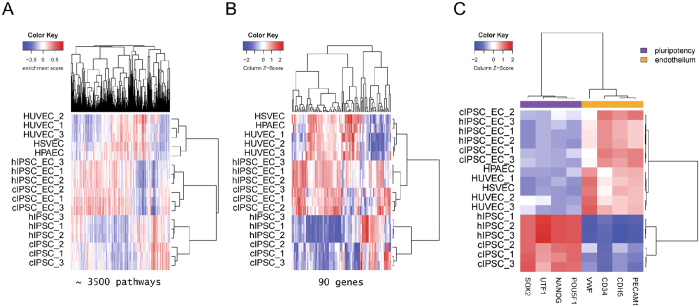
Differential pathway and gene expression activity across monkey and human ECs and IPSCs. (**A**) Heat map of differentially activated pathways. Columns represent gene ontology terms for biological processes and pathways, and rows are monkey and human ECs and IPSCs. Pathway enrichment scores were calculated on the whole transcriptome expression profiles for each sample using gene set variation analysis; blue indicates lower and red indicates higher pathway activity throughout the sample population. Hierarchical clustering of pathways and samples is based on complete linkage and Pearson correlation distance. (**B**) Heat map of genes annotated by the biological process of endothelium development (GO:0003158). Columns represent genes, and rows are monkey and human ECs and IPSCs. Column Z-score transformation was performed on log_2_ values for each gene, with blue denoting lower and red denoting higher expression levels according to the average expression level. Hierarchical clustering regarding the biological process shows clustering of monkey IPSC-derived ECs with high similarity to human IPSC-derived ECs and primary human ECs. (**C**) Heat map of marker gene panels for pluripotency and endothelial cells of the same samples as in (**B**). Columns represent genes and rows are samples. Column Z-score transformation was performed on log_2_ values for each gene. Hierarchical clustering of genes and samples is based on complete linkage and Pearson correlation distance.

First, we compared the transcriptomes of monkey and human IPSCs, IPSC-derived endothelial cells and primary human ECs (HUVEC, HSVEC, HPAEC) using RNA sequencing data. Principal component analysis (PCA) of transcriptome-wide expression profiles and unsupervised hierarchical clustering of ~3500 pathways with increased or decreased activity revealed three distinct clusters of cell types: IPSCs, IPSC-derived ECs, and primary human ECs (Fig. 3A and Supplementary Figure S3A, Supplementary Table 1). Strikingly, stem-cell derived ECs from monkeys were highly similar to human IPSC-derived ECs, which clustered tightly together. This finding was further confirmed by the fact that only very few biological processes were significantly different between monkey and human ECs, such as, for example, the response to vitamin D (GO:0033280) (Supplementary Figure S3B). Moreover, a biological pathway analysis showed that monkey and human IPSC-derived ECs exhibited significant over-representations of genes related to angiogenesis (GO:0001525), blood vessel development (GO:0001568) and endothelium development (GO:0003158) (Supplementary Figure S3B). With respect to these properties, monkey and human IPSC-derived ECs are highly similar to primary human ECs and are distinct to IPSCs, as shown by the hierarchical clustering of the expression profiles of genes for endothelium development (GO:0003158) and marker genes for endothelial cells in contrast to pluripotency (Fig. 3B,C). Taken together, the transcriptome-wide analysis of the pathway and gene expression profiles demonstrated that monkey and human IPSC-derived ECs are highly similar to each other and are similar to primary human ECs.

**Figure 4:**
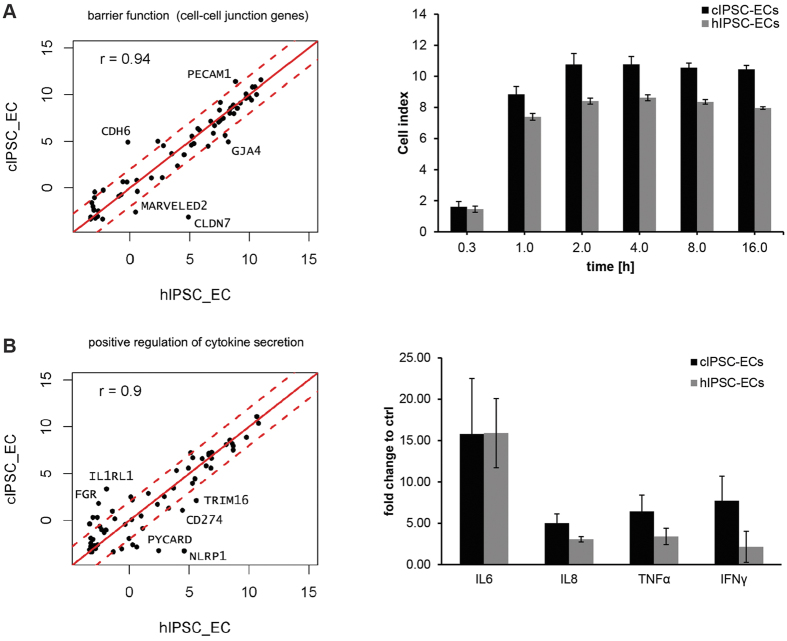
Monkey and human IPSC-derived endothelial cells as an *in vitro* model for inter-species comparisons. (**A**) Left: Scatterplot of barrier function genes involved in cell-cell-junction formation. Gene expression values for cIPSC-ECs compared to hIPSC-ECs are shown as the mean log_2_-transformed fragments per million mapped fragments (FPM + 0.1) of 3 replicates. Dashed lines indicate a fourfold cut-off in expression level difference. The Pearson correlation coefficient (r) between samples is shown at the upper left corner. Right: Impedance-based measurements of the EC monolayer reveal no species-specific differences between hIPSC-ECs and cIPSC-ECs. One of the 2 independent experiments performed is depicted (n = 4 technical replicates). Columns show the mean of 4 technical replicates +/− STD. (**B**) Left: Scatterplot of genes annotated by the biological process for positive regulation of cytokine secretion (GO:0050715). Gene expression values for cIPSC-ECs compared to hIPSC-ECs are shown as the mean log_2_-transformed fragments per million mapped fragments (FPM + 0.1) of 3 replicates. Dashed lines indicate the four-fold cut-off in expression level difference. The Pearson correlation coefficient (r) between samples is shown in the upper left corner. Right: Functional validation of gene expression analysis by analyzing cytokine release upon stimulation with IL1β. Twenty-four hours after treatment with 10 ng/ml IL1β, the release of proinflammatory cytokines was measured using multiplex ELISA. Columns show mean +/− STD of six biological replicates.

Next, we sought to analyze if a similarity in gene expression patterns translates to functional similarity, thereby focusing on key endothelial features, such as barrier formation and cytokine release. Genes involved in barrier formation and cell-cell junction organization were in general not significantly different in expression levels between monkey and human IPSC-derived ECs, which was also reflected by the Pearson correlation coefficient (r = 0.94) (Fig. 4A, Supplementary Table 2). Impedance measurements revealed that monkey and human IPSC-ECs formed and maintained a tight monolayer with a similar time course and efficiency (Fig. 4A). Likewise, monkey and human IPSC-derived ECs exhibited no significant differences in their biological processes, and for the genes annotated by the GO term “positive regulation of cytokine secretion (GO:0050715)”, the majority of gene expression levels were again highly correlated between species (Fig. 4B, Supplementary Table 3). Consistently, IPSC-ECs from both species exhibited similar release characteristics with respect to proinflammatory cytokines (IL6, IL8, TNFα, and IFNγ) upon treatment with IL1β. Importantly, we detected no species-specific differences regarding fold regulation of cytokine release. (Fig. 4B).

These data indicate that a similarity in gene expression patterns between IPSC-derived ECs from Cynomolgus monkeys and humans is reflected in similar functional features. Therefore, these cell models should be feasible for inter-species comparisons of endothelial functions with high translational values, e.g., in response to drug treatments causing endothelial activation or in contact with therapeutic antibodies.

## Discussion

IPSC-based *in vitro* systems represent valuable tools for studying cellular biology and developmental processes but also for drug screening or mechanistic studies for determining the mode of action or potential toxicities of drugs. In particular, the latter application plays an important role in pharmaceutical research, as *in vitro* cultures allow for the evaluation of relevant aspects of drug pharmacology and toxicity using simple, rapid and well controlled systems. Thus, IPSC technology is increasingly being explored to analyze and predict adverse drug reactions in the liver, heart or neurons. While such systems have been successfully developed for humans^22^,^23^,^24^ and are the focus of intensive validation^25^, similar models for NHPs are still lacking. However, due to the increasing complexity and species-specificity of novel drugs, such as therapeutic antibodies or RNA therapeutics (e.g., ASOs, siRNA), there is an increasing need to use NHPs for *in vivo* studies, which implies an increased need for predictive NHP *in vitro* systems.

Here, we present a NHP model for endothelial cells based on the directed differentiation of Cynomolgus monkey IPSCs. To ensure a reproducible and robust differentiation approach, we made use of a feeder-free culture system^15^ in combination with chemically defined differentiation conditions. This is in contrast to reported differentiation methods for NHP pluripotent stem cells, which generally use stem cell culture on feeder cells and differentiation methods based on parameters with high variability, such as embryoid body formation or the addition of serum needed for OP9 co-cultures^17^,^26^,^27^,^28^.

Thus, the presented method allows for the robust generation of NHP endothelial cells, representing a valuable alternative to other sources. While primary NHP ECs can be derived from animal tissue, they are linked to several constraints, such as donor variability and availability in required sufficient quantities e.g., for drug screening. IPSC-derived ECs not only can be generated in large scale, but IPSC technology also offers the ability to derive cells from different individuals, thus modeling the genetic variability amongst NHP populations.

Most importantly, endothelial cells can be obtained from human IPSCs using the same protocol with minor modifications^14^. Our data demonstrate that monkey IPSC-ECs are highly similar to the human counterpart regarding gene expression patterns and key functional features, such as inflammatory responses or barrier function. This shows the applicability of IPSC-derived ECs as a predictive *in vitro* model to perform inter-species comparison assays. Such systems could provide extremely useful tools in the development of novel drugs by enabling the evaluation of drug safety and efficacy in human and NHPs in the same cellular system. Moreover, the evaluation of drug efficacy in such *in vitro* systems will help to select the most potent drug candidates prior to *in vivo* studies, thus reducing the number of animal experiments.

Detailed analyses of EC differentiation from cIPSCs have revealed that the differentiation process is highly similar to vascular development during embryogenesis. Upon mesoderm commitment, cells are further directed towards the endothelial lineage by VEGF. Interestingly, using this protocol, mesoderm progenitors differentiate mainly into two cell types: ECs and vascular smooth muscle cells. This is in line with findings from hIPSCs, suggesting similarity in the differentiation process across species. However, when comparing the differentiation of cIPSCs and hIPSCs, differences in efficiency are observed. While human cells form ECs with an efficiency of 70%, cIPSCs subjected to the same method only differentiate into ECs with maximum efficiencies of 15%. This difference might be because all the growth factors and cytokines used are recombinant human proteins. Despite the high similarity between Cynomolgus monkeys and humans and the high degree of cross-reactivity observed for many factors, minor differences in protein sequence might influence binding kinetics to the corresponding receptors, resulting in different levels of activation or inhibition of the respective signaling pathway. As differentiation processes are regulated by a complex and sophisticated network of interacting signaling pathways, such minor differences might have a larger impact on aspects of the differentiation, such as efficiency. In the case of EC differentiation, enriching ECs by cell sorting can compensate for the lower differentiation efficiencies; however, it would be highly interesting to analyze if NHP-specific growth factors can markedly improve EC formation and proliferation.

## Methods

### Cell culture

All animal procedures were performed in accordance with the guidelines of the National Institutes of Health and approved by the Institutional Animal Care and Use Committee (IACUC) affiliated with Roche 340 Kingsland Street Nutley NJ 07110, USA (closed 2014). Cynomolgus IPSCs were established from kidney fibroblasts from a female, 14-year-old Mauritian Cynomolgus monkey using Sendai virus particles (CytoTune-iPS Sendai Reprogramming Kit, Thermo Fisher Scientific) harboring the Yamanaka factors (Oct4, Sox2, Klf4, and C-Myc)^29^. Five days post-transfection, cells were passed onto mitomycin C inactivated feeders at varying densities and cultured in hESC media (knock-out DMEM:F12 supplemented with 20% knock-out serum replacement, 0.1 mM non-essential amino acids, 2 mM L-glutamine, 0.1 mM 2-mercaptoethanol, and 8 ng/ml bFGF, all from Life Technologies). Twenty days post-transfection clones with ES-like morphologies were selected for further passaging. The cells were routinely passaged every 3–4 days by manual dissociation of colonies. After at least 10 passages, cIPSCs were adapted to feeder-free culture conditions based on Ono *et al*.^15^ with the modification that cells were cultured on Matrigel (BD Bioscience)-coated plates and that Y-27632 (Calbiochem) was used instead of Thiazovivin when the cells were in single cell suspension. The cells were passaged every 2–4 days using Gentle Cell Dissociation Reagent (Stem Cell Technologies). One clone was chosen for further experiments and used for all the following assays (cIPSC line A). The cIPSC line B was obtained from Ulrich Martin (Medical School Hannover) and has been characterized in another study^11^.

Human IPSCs were derived from neonatal foreskin fibroblasts (Lonza), as described above. After adaption to feeder-free culture conditions, the cells were cultured on Matrigel-coated plates in mTesR1 (StemCell Technologies) medium including supplements and were passaged once per week using GCD.

Human primary cells (from Lonza: HUVEC, HSVEC; from Cascade Biologics: HPAEC) were grown until confluency in EGM-2 medium (Lonza).

### Karyotyping

G-banding chromosomal analysis was performed by Cell Line Genetics.

### Endothelial differentiation

Endothelial differentiation was performed as described in Patsch *et al*.^14^. Briefly, IPSCs were detached using Accutase (Stem Cell Technologies) and seeded on growth factor-reduced Matrigel (BD Bioscience) in the corresponding stem cell medium (mTesR1 for hIPSCs, MT medium for cIPSCs) supplemented with 10 μM Rock Inhibitor Y-27632. After 24 hours, the cells were washed with PBS and covered with priming medium consisting of N2B27 supplemented with 25 ng/ml BMP4 (Gibco) and CP21 (Roche) at the indicated concentrations. After the priming period (between 3–4 days), the cells were washed, and the medium was switched to induction medium consisting of StemPro34-SFM complete (Life Technologies) supplemented with 200 ng/ml VEGF (Peprotech) and 2 μM forskolin (Roche). The induction medium was changed every other day.

At day 6 of differentiation, ECs were dissociated with Accutase or 0.05% trypsin-EDTA and MACS-separated using CD144 MicroBeads (Miltenyi Biotec) and an autoMACSpro (Miltenyi Biotec). Positive-sorted cells were replated on human fibronectin (Sigma-Aldrich)-coated dishes in EC expansion medium consisting of StemPro-34 SFM supplemented with 50 ng/ml VEGF. For functional assays, the cells were cultured in EBM medium supplemented with the EGM2 bullet kit (Lonza).

### Immunofluorescence staining

Cells were fixed with 4% PFA and permeabilized with 0.1% TritonX (Sigma) in PBS (with Ca^2+^ and Mg^2+^). Blocking was performed using SuperBlock solution (Thermo Fisher Scientific) supplemented with 0.1% TritonX. Cells were stained with the following primary antibodies: anti-OCT4 (Santa Cruz Biotechnology), NANOG (Santa Cruz Biotechnology), anti-SOX2 (Merck Millipore), anti-VE-Cad (R&D Systems), anti-PECAM (R&D Systems), anti-ICAM1 (R&D Systems), anti-vWF (Dako), and anti-Brachyury (R&D Systems). Subsequently, the cells were washed and stained with secondary antibodies conjugated to Alexa488, Alexa555, and Alexa647 (all Molecular Probes). Nuclei were stained with Hoechst 1:1000 (Molecular Probes). The cells were imaged using an Axiovert microscope (Zeiss). Images were analyzed using ImageJ software.

### Image-based quantification of ICAM1 expression

For quantification of ICAM1 expression an Operetta High-Content Imaging System (Perkin Elmer) was used and images were analyzed using the Harmony High-Content Imaging and Analysis Software (Perkin Elmer).

### Flow cytometry

For flow cytometry, the cells were stained for 15 min at 4 °C, in the dark in 100 μl MACS running buffer (Miltenyi Biotec) containing the antibodies detecting CD144 (BD Bioscience), CD31 (Biolegend), CD140b (BD Bioscience), CD309/KDR (Miltenyi Biotec), and CD45 (BD Bioscience). Afterwards, cells were washed with MACS running buffer and resuspended in 500 μl MACS running buffer. Flow cytometry was performed using a BD FACS Canto, and the data were analyzed with FlowJo software (Tree Star).

### Tube formation

Growth factor-reduced Matrigel (250 μL) was aliquoted into each well of a 24-well plate and incubated for 60 min at 37 °C to allow the gel to solidify. Then, 50,000 ECs were seeded onto the matrix in StemPro34-SFM complete containing 100 ng/ml VEGF and cultured for 24 h at 37 °C until image acquisition. To inhibit angiogenesis, 10 μM sulforaphane (Sigma S4441) or 2 μg/ml anti-VEGF^18^ was added to the medium.

### Uptake of Dil-Ac-LDL

Cells were incubated with 2.5 μg/mL DiI-Ac-LDL-Alexa488 (Molecular Probes) for 4 h at 37 °C. Thereafter, the cells were washed three times with PBS, covered with fresh medium and imaged using an Axiovert 200 microscope (Zeiss).

### Real time impedance measurement

Cell growth behavior was continuously monitored using a Real Time Cell Analyzer (xCELLigence, Roche). For time-dependent cell response profiling, 50 μl of cell culture medium was added to the fibronectin coated 96-well E-plates to obtain background readings followed by the addition of 50 μl of cell suspension. The E-plates containing the cells (30,000 cells/96 wells) were placed on the reader in the incubator for continuous recording of impedance, as reflected by the cell index (CI). The cells were monitored every 15 minutes for 4 hours and subsequently every hour for another 14 hours. The CI curves are displayed as the average of 4 replicates +/− standard deviation.

### Cytokine release

Endothelial cells were seeded at 30,000 cells/well in 96-well plates. After cell attachment (24 hours), the cells were treated with 10 ng/ml IL1β (Sigma) for 24 hours. The supernatants were collected and analyzed using a species-specific Ciraplex assay kit (Aushon). Cytokine release was calculated as the fold-change compared to non-treated cells, and six replicates were analyzed.

### RNA-sequencing

RNA was isolated from Cynomolgus monkey or human cells using QIAzol for cell lysis and the miRNeasy Mini kit combined with DNase treatment on a solid support for RNA extraction (Qiagen). RNA quality with RIN values > 9.7 was determined by electrophoresis on Agilent RNA 6000 Nano Bioanalyzer microfluidic chips. Template DNA molecules suitable for sequencing were prepared from 500 ng of total RNA using the TruSeq Stranded mRNA Library Preparation Kit (Illumina) according to the manufacturer’s instructions. After 12 cycles of PCR amplification, the size distribution of the barcoded DNA libraries was estimated by electrophoresis on Agilent High Sensitivity Bioanalyzer microfluidic chips. The minimum sizes of the amplified libraries were determined as >200 nucleotides and the average size was ~300 nucleotides. Libraries were quantified using the KAPA Library Quantification Kit (Kapa Biosystems). Libraries were pooled at equimolar concentrations, spiked with 1% PhiX control library, and diluted to 11 pM prior to loading onto the flow cell of an Illumina HiSeq 2500 instrument (Illumina) for both clustering and sequencing. Libraries were extended and bridge amplified to create single sequence clusters using the HiSeq PE Cluster Kit v4 cBot HS (Illumina). The flow cell carrying amplified clusters were then sequenced in high output run mode with 51 cycles for read 1, 7 cycles for the barcode index, and 51 cycles for read 2 using the HiSeq SBS Kit v4 chemistry (Illumina). 2×50-bp paired-end reads were generated with ~30 million read pairs per sample. Real time image analysis and base calling was performed using the HiSeq 2500 with the HiSeq Control Software v2.2.37. CASAVA software version 1.8.2 was used for de-multiplexing and for the production of the FASTQ sequence files.

To generate gene level read counts, matched pairs of reads from the FASTQ files were aligned to the Cynomolgus monkey genome version 5.0^1^ for monkey samples or to the human genome (GRCh38/hg38) for human samples using Bowtie2/HTSeq. Read counts from the annotated genes were library-size normalized and modelled by DESeq2 using a negative binomial distribution; differential expression was quantified by generalized linear models implemented in the package. Gene set analysis was performed according to the gene set enrichment analysis (GSEA) method from the Broad Institute (http://www.broadinstitute.org/gsea), and a gene set database comprised gene ontology (GO) terms for the biological processes (www.geneontology.org). For the entire GSEA, a gene set size filter from 15 to 500 genes per GO term was applied, 1000 phenotype permutations were performed, and log2-fold change values were used as a metric to create the ranked list of genes. To estimate variations in pathway activity over the sample population, a gene set variation analysis (GSVA) was performed^30^. This method utilizes the gene expression profile of each sample to estimate the activity of pathways across the overall sample population in an unsupervised manner. Pathways with a gene set size from 15 to 500 genes were taken into account for the gene set database of the GO biological processes. The data were visualized using R software for statistical computing and graphics (http://www.r-project.org). The RNA-sequencing data from this study have been deposited at the NCBI Gene Expression Omnibus (http://www.ncbi.nlm.nih.gov/geo) under accession number GSE84385.

## Additional Information

**How to cite this article**: Thoma, E. C. *et al*. Establishment of a translational endothelial cell model using directed differentiation of induced pluripotent stem cells from Cynomolgus monkey. *Sci. Rep*. **6**, 35830; doi: 10.1038/srep35830 (2016).

## Supplementary Material

Supplementary Information

Supplementary Table S1

Supplementary Table S2

Supplementary Table S3
