# Supplementary material for: Establishment of a translational endothelial cell model using directed differentiation of induced pluripotent stem cells from Cynomolgus monkey
Source: Sci Rep. 2016 Oct 25;6:35830. doi: 10.1038/srep35830 (PMC5078800; doi:10.1038/srep35830)

Supplementary Information

Establishment of a translational endothelial cell model using directed differentiation of induced pluripotent stem cells from Cynomolgus monkey

Eva C Thoma[1*], Tobias Heckel[1*], David Keller[1], Nicolas Giroud[1], Brian Leonard[1], Klaus Christensen[1], Adrian Roth[1], Cristina Bertinetti-Lapatki[1], Martin Graf[1], Christoph Patsch[1**]

*: Equal contribution

**: Corresponding author:

Christoph Patsch

F. Hoffmann-La Roche Ltd.

Grenzacherstrasse 124

4070 Basel, Switzerland

Email: christoph.patsch@roche.com

Phone: +41 61 68 82104

1: Roche pRED (Pharmaceutical Research and Early Development), Roche Innovation Center Basel, F.Hoffmann-La Roche Ltd., Basel, Switzerland

Supplementary Figure 1: Characterization of cIPSCs grown in feeder-free conditions. (A) Phase contrast image of cIPSCs. (B-D) Immunostaining shows the expression of pluripotency markers OCT4, NANOG, and SOX2 in cIPSCs. Scale bars: 100 µm. (E) Karyotype analysis of cIPSCs reveals normal karyotype.

Supplementary Figure 2: Endothelial differentiation of cIPSCs. (A) Optimization of differentiation protocol. The effect of different concentrations of CP21, the length of priming phase, and the initial seeding densities were analyzed by flow cytometry at day 6 of differentiation. Efficiency was evaluated by determining the percentage of CD144+/CD140b- cells (left) and CD31+ cells (right). (B) Reproducibility of the differentiation protocol using cIPSC lines derived from different animals. Columns show mean +/- STD of three independent experiments. (C) Enrichment of endothelial cells using magnetic-activated cell sorting of CD144 positive cells. (D) Heat map of biological processes (Gene Ontology terms) significantly over- or under-represented during the differentiation time course of cIPSCs. The heat map shows directed $-\log_{10}$ family-wise error rate (FWER) corrected p-values for upregulated (red) and downregulated (blue) biological processes. White indicates no change. Upregulated and downregulated biological processes were determined by gene set enrichment analysis of whole transcriptome differential gene expression signals of cIPSCs (day 0) in comparison to differentiated cells. Most significantly enriched biological processes across all time points were determined by a p-value of 0.001, which corresponds to an absolute score of 3 on this scale. Hierarchical clustering of biological processes and samples is based on complete linkage and Pearson correlation distance.

Supplementary Figure 3: Global transcriptome and pathway comparison between monkey and human IPSC-derived endothelial cells. (A) Principal component projections of monkey and human transcriptomes colored by cell type. The variability of the data set along principal component 1 is 62% and along principal component 2 is 19%. EC: endothelial cell, HUVEC: human umbilical vein EC, HSVEC: human saphenous vein EC, HAEC: human aortic EC. (B) Gene set enrichment analysis of significantly over- or under-represented biological processes (gene ontology terms) in endothelial cells. Bar charts show the top 20 biological processes ranked according to family-wise error rate corrected p-values; a p-value of 0.001 corresponds to an absolute score of 3 on this scale. Upregulated biological processes are shown in red and downregulated in blue. Top upregulated biological processes shared between monkey and human cells are indicated in magenta. Upregulated and downregulated biological processes were determined by the gene set enrichment analysis of the whole

transcriptome differential gene expression signals of monkey or human induced pluripotent stem cells, respectively, in comparison to differentiated cells, and of monkey in comparison to human endothelial cells.

Supplementary Table 1: GSVA Enrichment Scores: Pathway enrichment scores for biological processes with increased or decreased activity were determined by the gene set variation analysis of the whole transcriptome gene expression signals interrogating 3539 gene ontology terms; cIPSC: Cynomolgus monkey induced pluripotent stem cells, hIPSC: human IPSCs, cIPSC-EC: cIPSC-derived endothelial cells (EC), hIPSC-EC: hIPSC-derived EC, HUVEC: human umbilical vein EC, HSVEC: human saphenous vein EC, HPAEC: human pulmonary artery EC.

Supplementary Table 2: Differentially expressed genes for "barrier function (cell-cell junction genes)": Log_2 Fold-change values were calculated using DESeq2 for cIPSC-ECs in comparison to hIPSC-ECs (n = 3 replicates per species). Genes were selected by GO term, GO:0045216.

Supplementary Table 3: Differentially expressed genes for "positive regulation of cytokine secretion": Log_2 Fold-change values were calculated using DESeq2 for cIPSC-ECs in comparison to hIPSC-ECs (n = 3 replicates per species). Genes were selected by GO term, GO:0050715.

Supplementary Figure S1

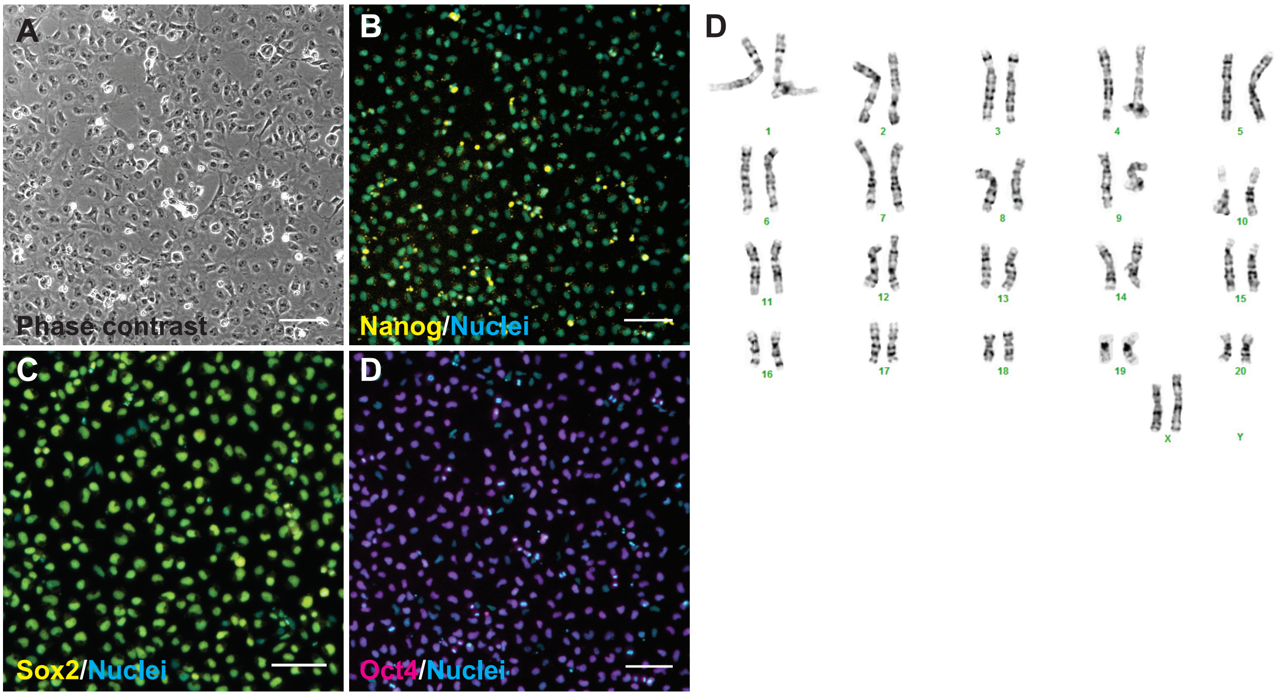

Supplementary Figure S2

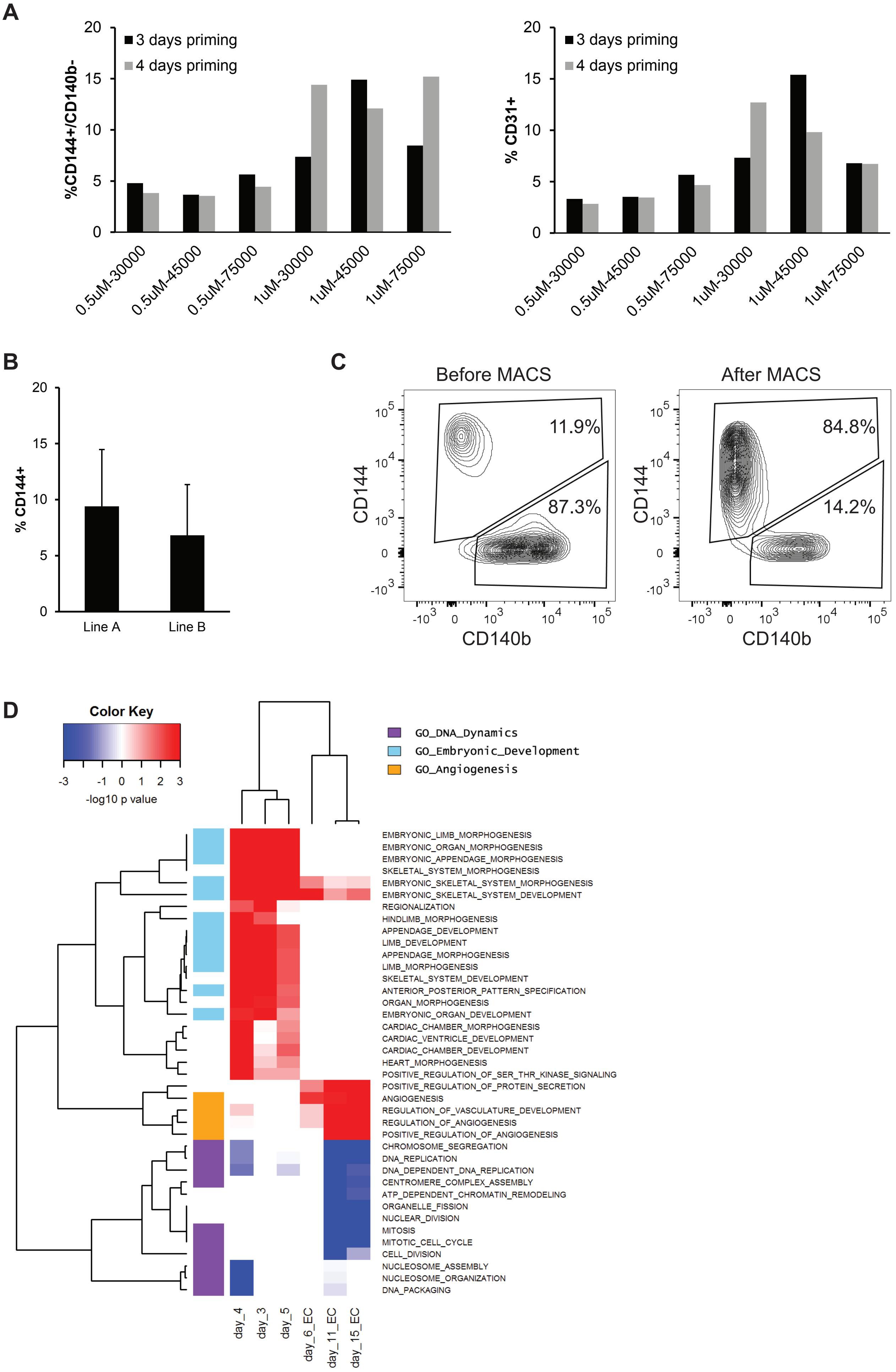

Supplementary Figure S3

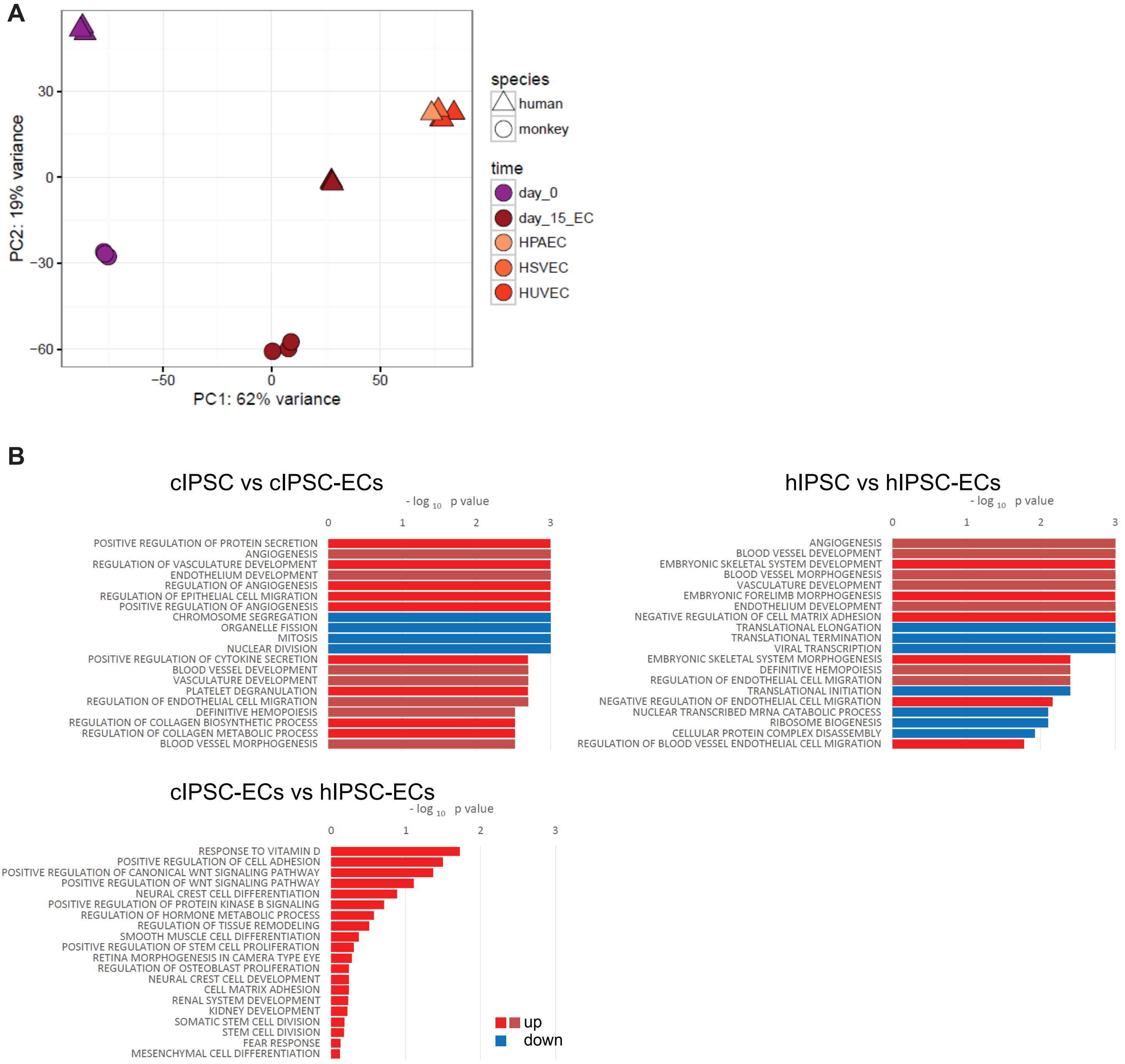

Supplement: Supplementary Information [file srep35830-s1.pdf]
